# Cardiovascular disease incidence prediction by machine learning and statistical techniques: a 16-year cohort study from eastern Mediterranean region

**DOI:** 10.1186/s12911-023-02169-5

**Published:** 2023-04-19

**Authors:** Kamran Mehrabani-Zeinabad, Awat Feizi, Masoumeh Sadeghi, Hamidreza Roohafza, Mohammad Talaei, Nizal Sarrafzadegan

**Affiliations:** 1 Cardiovascular Research Center, Cardiovascular Research Institute, Isfahan University of Medical Sciences, Isfahan, Iran; 2 Biostatistics and Epidemiology Department, School of Health, Isfahan University of Medical Sciences, Isfahan, Iran; 3 Cardiac Rehabilitation Research Center, Cardiovascular Research Institute, Isfahan University of Medical Sciences, Isfahan, Iran; 4 Wolfson Institute of Population Health, Barts and The London School of Medicine and Dentistry, Queen Mary University of London, London, UK; 5 School of Population and Public Health, Faculty of Medicine, University of British Columbia, Vancouver, Canada

**Keywords:** Cardiovascular, Machine learning, Statistical models, Cohort study, Eastern Mediterranean region, Feature selection, Missing values

## Abstract

**Background:**

Cardiovascular diseases (CVD) are the predominant cause of early death worldwide. Identification of people with a high risk of being affected by CVD is consequential in CVD prevention. This study adopts Machine Learning (ML) and statistical techniques to develop classification models for predicting the future occurrence of CVD events in a large sample of Iranians.

**Methods:**

We used multiple prediction models and ML techniques with different abilities to analyze the large dataset of 5432 healthy people at the beginning of entrance into the Isfahan Cohort Study (ICS) (1990–2017). Bayesian additive regression trees enhanced with “missingness incorporated in attributes” (BARTm) was run on the dataset with 515 variables (336 variables without and the remaining with up to 90% missing values). In the other used classification algorithms, variables with more than 10% missing values were excluded, and MissForest imputes the missing values of the remaining 49 variables. We used Recursive Feature Elimination (RFE) to select the most contributing variables. Random oversampling technique, recommended cut-point by precision-recall curve, and relevant evaluation metrics were used for handling unbalancing in the binary response variable.

**Results:**

This study revealed that age, systolic blood pressure, fasting blood sugar, two-hour postprandial glucose, diabetes mellitus, history of heart disease, history of high blood pressure, and history of diabetes are the most contributing factors for predicting CVD incidence in the future. The main differences between the results of classification algorithms are due to the trade-off between sensitivity and specificity. Quadratic Discriminant Analysis (QDA) algorithm presents the highest accuracy (75.50 ± 0.08) but the minimum sensitivity (49.84 ± 0.25); In contrast, decision trees provide the lowest accuracy (51.95 ± 0.69) but the top sensitivity (82.52 ± 1.22). BARTm.90% resulted in 69.48 ± 0.28 accuracy and 54.00 ± 1.66 sensitivity without any preprocessing step.

**Conclusions:**

This study confirmed that building a prediction model for CVD in each region is valuable for screening and primary prevention strategies in that specific region. Also, results showed that using conventional statistical models alongside ML algorithms makes it possible to take advantage of both techniques. Generally, QDA can accurately predict the future occurrence of CVD events with a fast (inference speed) and stable (confidence values) procedure. The combined ML and statistical algorithm of BARTm provide a flexible approach without any need for technical knowledge about assumptions and preprocessing steps of the prediction procedure.

## Introduction

Cardiovascular disease (CVD) is a leading cause of global death since 1980 [1]. World Health Organization (WHO) reports noted that each year 17.9 million people die from CVD, accounting for approximately 32% of worldwide deaths, and 75% of them occur in low and middle-income countries [2]. Coronary artery disease, cerebrovascular disease, peripheral arterial disease, rheumatic heart disease, congenital heart disease, deep vein thrombosis, pulmonary embolism, acute myocardial infarction, and stroke are common types of CVD [2].

Some CVD risk factors such as age, gender, ethnicity, and family history are non-modifiable; However, leading modifiable risk factors include high blood pressure, diabetes, dyslipidemia, obesity, low or lack of physical activity, unhealthy diet, stress, and smoking [3–7]. Currently, policymakers in the area of CVD prevention and control guidelines recommended the use of CVD risk prediction models in order to determine and highlight the high-risk people that early interventions could lead to a reduction in CVD incidence. Accordingly, risk prediction models using traditional statistical methods as well as machine learning approaches have been commonly used in this subject area. Prediction models based on machine learning algorithms are robust against common limitations such as non-linearity, multicollinearity, interaction, and particularly complexities available in large datasets in traditional statistical models [8–10]. Therefore, it is expected that prediction models based on machine learning algorithms will show higher predictive performance compared to traditional statistical methods [11–16], although there are controversies about the superiority of these models compared to each other [17, 18].

The CVD events rates vary across the different regions of the globe, so investigating the risk factors in each region can help to find the main specific causes of CVD in that region. The results of such regional specific studies help the policymakers to adopt the proper CVD prevention and control programs [19]. Despite the high CVD prevalence and incidence in developing countries, studies on establishing risk prediction models in these countries are scarce. The majority of CVD prediction models using ML techniques have been conducted in developed countries [13, 20–24]. Less adoption of ML techniques in developing countries can be for three reasons: (I) availability of comprehensive and accurate datasets in the CVD field [25, 26]; (II) financial difficulties leading to only a few research centers in these countries being able to purchase high specification computers to run ML techniques on large datasets [27]; (III) lack of expertise in the ML field [28].

This study adopts the most popular ML algorithms used in CVD prediction studies, including k-Nearest Neighbors (kNN), Support Vector Machine (SVM), Decision Trees (DT), Random Forest (RF), Artificial Neural Network (ANN), and Gradient Boosting Machine (GBM) to develop suitable and efficient prediction models for predicting the future occurrence of CVD events based on the comprehensive set of risk factors in the framework of the long-term Isfahan Cohort Study (ICS), a population-based cohort in the eastern Mediterranean region, Iran. This study also aimed to identify the most efficient predictors of future CVD incidence in participants who were healthy at the entrance to the ICS in order to find a high-risk group for early CVD events. This study also attempted to compare the predictive abilities of the machine learning modeling approach with traditional statistical methods.

## Materials and methods

### Study design and participants

This study is a secondary analysis of the ICS dataset; An ongoing longitudinal population-based prospective cohort study [29]. This cohort started in 2001 in three central cities of Iran (Isfahan, Najafabad, and Arak). According to Iran’s census in 2016, Isfahan is the third most populated city with a population of 2ˏ243ˏ249, Najafabad and Arak had 319ˏ205 and 591ˏ756 populations, respectively. In ICS, 6323 participants were recruited based on multistage random sampling from January 2 through September 28, 2001. The inclusion criteria were: being Iranian, aged 35 or older, mentally competent, and not pregnant. The exclusion criteria were: having any CVD events at baseline. In this study, among the 6323 participants, 5432 participants which had at least one follow-up were entered. The ICS study was performed by Isfahan Cardiovascular Research Center (ICRC), a WHO-collaborating center (https://apps.who.int/whocc/Search.aspx). All participants were interviewed by trained health professionals and data were recorded into proper questionnaires and checklists. Every five years, all participants had follow-up visits for full medical examination and blood sampling for further evaluations. Also, twice a year all participants were evaluated by phone calls for tracking the occurrence of certain predefined events. Detailed information about ICS has been provided in the previously published report [29].

All available data on study participants in 2001 was considered as potential risk factors for the occurrence of any CVD events until 2017 as the response variable. The current secondary study protocol was reviewed and approved by the ethics committee of Isfahan university of medical sciences (approval number. IR.MUI.MED.REC.1400.493).

### Risk factors

A comprehensive dataset containing more than 1000 variables, basic and clinical characteristics of study participants, collected through data collection by ICS, was considered as a source of potential predictors of CVD events. It includes the following categories: Sociodemographic characteristics, including age, gender, and education level (classified as 0–5 years, 6–12 years, and > 12 years). Cardio-metabolic factors include Body Mass Index (BMI), Systolic Blood Pressure (SBP), Diastolic Blood Pressure (DBP), High-Density Lipoprotein (HDL), Low-Density Lipoprotein (LDL), and triglyceride. Lifestyle factors, including smoking, physical activity, dietary habits and intake. History of diabetes was defined according to participants’ self-reports, and they were diagnosed with diabetes mellitus when Fasting Blood Sugar (FBS) ≥ 126 mg/dL or by using anti-diabetic agents [30]. Generally, the dataset contains more than 1000 variables. By excluding the variables with more than 90% of missing values, 515 variables remained; Among them, 336 variables were complete without any missing data, 49 variables had less than 10% missing values, and the remaining 130 variables had more than 10% missing values.

### Study outcome

The response variable in the current study was considered as any diagnosis of CVD events until 2017, which includes: fatal and non-fatal myocardial infarction, fatal and non-fatal stroke, sudden cardiac death, and unstable angina. The decision about CVD events diagnosis was confirmed by a special panel including four expert cardiologists and an expert neurologist [29]. Among all 5432 participants, CVD events occurred for 819 participants (15.08%) in the follow-up period; Hence, the response variable is imbalanced relevant techniques and evaluation metrics should be used during modeling.

**Fig. 1:**
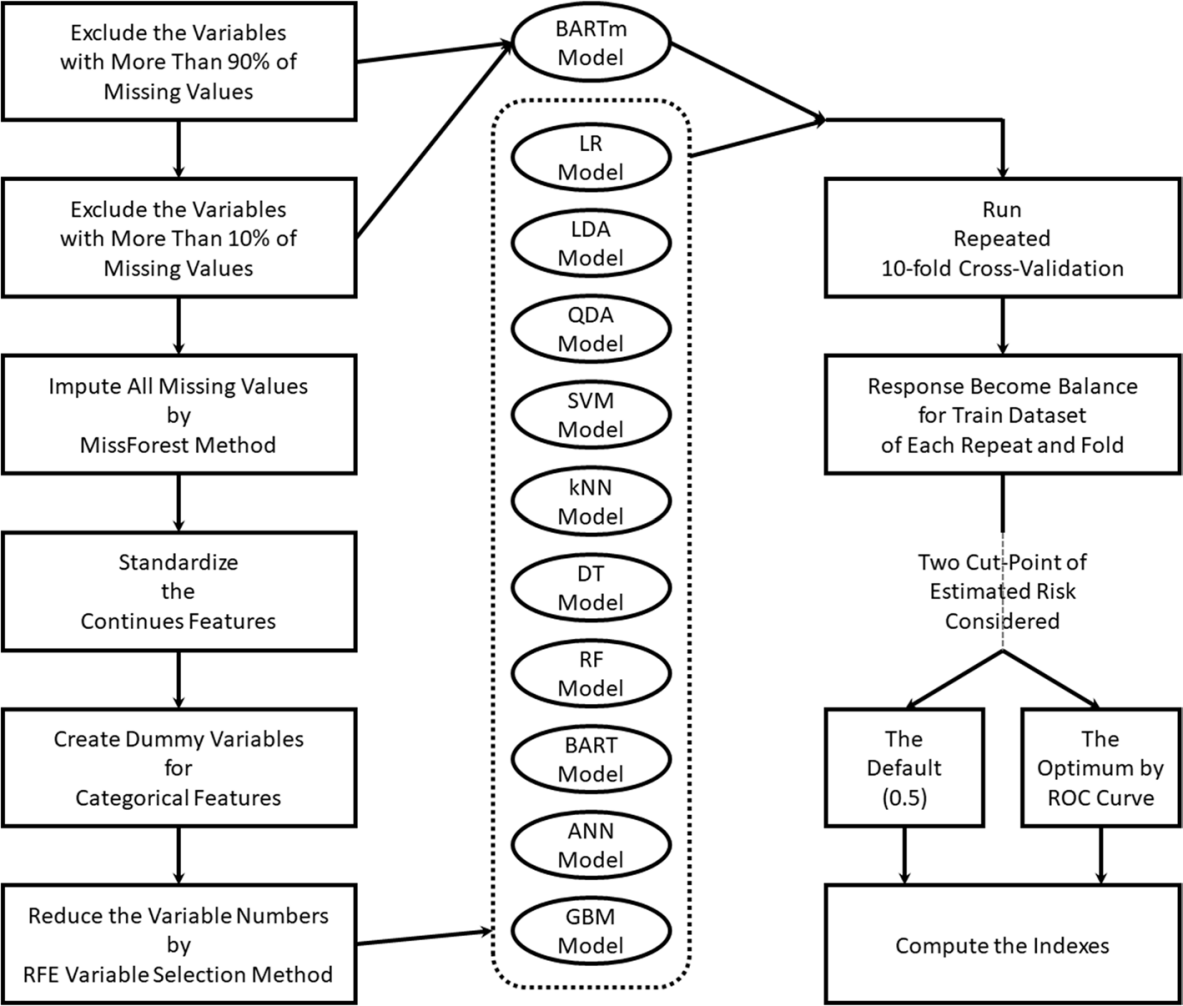
The flow of the data analysis process

Figure 1 presents the flow of the data analysis process that was carried out in this study. This has been described in detail in the following sections.

### Classification algorithms implementation

Several ML algorithms have been utilized for CVD incidence prediction but there is no unique model with the highest predictive ability in all situations [15]. A meta-analysis on 344 studies showed that the SVM and GBM have the highest predictive ability [31]. A review article in 2022 indicated that RF and ANN have the best predictive performance [32]. So, in this study, the various supervised classical statistical and machine learning classification models were used by considering their predictive power and popularity, including Logistic Regression (LR), Linear Discriminant Analysis (LDA), Quadratic Discriminant Analysis (QDA), SVM, kNN, DT, RF, Bayesian Adaptive Regression Trees (BART), missing incorporated to attributes-within BART (BARTm), ANN and GBM.

All models run according to the same procedure except BARTm. The BARTm model has a combined statistical and ML algorithm that makes it capable of accurately classifying data even with 90% of missing values, without any imputation [33]. So, the BARTm model was implemented on the dataset with two missing value scenarios: (I) all variables with up to 90% of missing values were considered (515 variables); (II) only those variables with up to 10% missing values were considered (385 variables). These two model verifications of the BARTm model were denoted by BARTm.90% and BARTm.10%, respectively.

The grid search cross-validation techniques were applied to tune the hyper-parameters of ML algorithms that determine the optimal values to achieve higher accuracy. The tuned parameters of each algorithm and their optimal values are presented in Table 1.

**Table 1:** Hyper-parameters of applied ML algorithms in this study

Algorithm	Parameters	Optimal Value
**BARTm, BART**	Number of trees	50
	Prior probability (k)	2
**kNN**	Number of neighbors	27
**SVM**	Gamma parameter	0.125
	Cost of constraint violation	1
**DT**	Complexity parameter	0.01
**RF**	Number of candidate variables at each split	2
**ANN**	Number of the units in the hidden layer	2
	Decay weight parameter	0.1
**GBM**	Number of iterations	150

### Dataset preprocessing

Dataset preprocessing in the ML algorithms leads to better model prediction performance. Because of BARTm model’s efficiency, it was implemented on a dataset without any preprocessing steps. The following preprocessing steps have been conducted for implementing the other models, which cannot manage the missing values.

In the first step, the variables with more than 10% missing values were excluded, so 385 variables remained. MissForest procedure was used to impute the missing values of the remaining 49 variables with up to 10% missing values. However, the MissForest procedure as a nonparametric RF-based imputation of missing value is time-consuming but at the same time, it outperforms compared to other imputation approaches and provides more accurate imputation [34]. In the next step, each continuous variable was standardized by centering with its minimum and scaling with its range; Also, from categorical variables having more than two categories, dummy variables were created.

Before conducting the model training process, in order to make optimal applicability of all used models and compare their prediction ability with each other, it is necessary to reduce the number of predictors. Recursive Feature Elimination (RFE) method was used to select the most informative variables or dimension reduction for prediction. Although it has an intensive computational burden, it is one of the more effective dimensional reduction procedures. It considers a wide range of patterns and correlations in the dataset and then chooses the most contributing variables for prediction [35]. Therefore, after applying RFF, each used classification model will be applied to fewer informative predictors. This feature selection step is one of the main benefits of ML that makes the conventional statistics models applicable to large datasets [36].

For obtaining more reliable results during the train and test models specification, each model was run under a repeated 10-fold cross-validation algorithm. The incidence rate of CVD events in this study sample was 15.08%, so the two response levels are imbalanced. Therefore, for each training dataset in each repeat and fold, the random oversampling technique was performed.

### Statistical analysis and models evaluation metrics

After each 10-fold cross-validation, for converting the predicted risk probability to binary classes based on all used classification algorithms, two options including predefined default cut-points in each algorithm and the optimal cut-point recommended by Precision-Recall Curve (PRC) that maximizes the F-score were adopted.

The following metrics were considered to evaluate and compare the predictive power of applied models: accuracy, balanced accuracy, sensitivity, specificity, Area Under the Receiver Operating Characteristic Curve (AUROC), Area Under the Precision-Recall Curve (AUPRC), and normalized Matthews Correlation Coefficient (nMCC).

We reported continuous data as mean ± Standard Deviation (SD) and categorical data as numbers (percentages). Independent t-test and chi-square test were used to compare continuous and categorical risk factors between participants who experienced CVD events with other ones, respectively.

All analyses were conducted in R statistical software version 4.1.1 [37] by using the following packages: bartMachine [38] for BARTm and BART models, MASS [39] for LDA and QDA models, caret [40] for kNN model and also RFE procedure, e1071 [41] for SVM model, rpart [42] for DT model, randomForest [43] for RF model, nnet [39] for ANN model, gbm [44] for GBM model, missForest [45] for imputing missing values, pROC [46] for ROC analysis and PRROC [47] for precision-recall analysis.

## Results

Overall, the mean age of participants at baseline was 50.49 ± 11.49 years, and 2697(51.00%) were female. During the 16-year follow-up, 819 (15.08%) experienced occurrences of any CVD events. Table 2 presents the basic characteristics of the 5432 included participants of ICS in this study in two groups of participants with and without experiencing CVD events.

**Table 2:** Basic characteristics of ICS study in the CVD and non-CVD groups

**Variable**	**CVD** **(*n* = 819)**	**Non-CVD** **(*n* = 4613)**	*p*-value*
**Sociodemographic Factors**			
**Age**	56.59 ± 11.62	49.25 ± 11.06	< 0.001
**Gender**			< 0.001
**Female**	407 (15.09%)	2290 (84.91%)	
**Male**	486 (18.75%)	2106 (81.25%)	
**Education**			0.003
**0–5 year**	675 (18.02%)	3071 (81.98%)	
**6–12 year**	170 (14.01%)	1043 (85.99%)	
**≥ 13 year**	48 (14.55%)	282 (85.45%)	
**Cardiometabolic Factors**			
**BMI**			< 0.001
**Normal/Underweight**	267 (13.59%)	1697 (86.41%)	
**Overweight**	399 (18.80%)	1723 (81.20%)	
**Obese**	219 (18.54%)	962 (81.46%)	
** Waist Circumference (cm)**	97.73 ± 12.35	94.19 ± 12.84	< 0.001
** Systolic Blood Pressure (mmHg)**	131.86 ± 23.57	119.41 ± 19.64	< 0.001
** Diastolic Blood Pressure (mmHg)**	82.57 ± 12.49	77.48 ± 11.07	< 0.001
** High-Density Lipoprotein (mg/dL)**	46.94 ± 10.53	46.90 ± 10.32	0.920
** Low-Density Lipoprotein (mg/dL)**	137.54 ± 46.37	127.12 ± 42.55	< 0.001
** Triglyceride (mm/dL)**	216.47 ± 115.18	185.82 ± 99.24	< 0.001
** Total Cholesterol (mg/dL)**	227.77 ± 56.20	211.16 ± 50.82	< 0.001
**Lifestyle Factors**			
**Ever Smoking**			0.102
**Yes**	163 (18.78%)	705 (81.22%)	
**No**	728 (16.49%)	3687 (83.51%)	
** Global Dietary Score**	0.98 ± 0.27	1.03 ± 0.24	< 0.001
** Total Daily Physical Activity**	799.80 ± 556.32	895.65 ± 543.69	< 0.001

^*^Resulted from independent samples t-test or chi-squared test

The RFE procedure recommends only 8 variables as an optimal subset for this study. Descriptive statistics of these 8 variables across CVD events categories are presented in Table 3.

**Table 3:** Most contributing risk factors for CVD prediction

**Variable**	**CVD** **(*n* = 819)**	**Non-CVD** **(*n* = 4613)**	*p*-value*
**Age**	56.59 ± 11.62	49.25 ± 11.06	< 0.001
**Systolic Blood Pressure (mmHg)**	131.86 ± 23.58	119.41 ± 19.64	< 0.001
**Fasting Blood Sugar (mg/dL)**	99.48 ± 46.89	86.26 ± 28.07	< 0.001
**Two-hour Postprandial Glucose (mg/dL)**	119.52 ± 64.63	104.67 ± 43.43	< 0.001
**History of Heart Disease**			< 0.001
**Yes**	148 (41.69%)	207 (58.31%)	
**No**	745 (15.10%)	4189 (84.90%)	
**History of High Blood Pressure**			< 0.001
**Yes**	247 (35.90%)	441 (64.10%)	
**No**	646 (14.04%)	3955 (85.96%)	
**History of Diabetes**			< 0.001
**Yes**	146 (39.14%)	227 (60.86%)	
**No**	747 (15.20%)	4169 (84.80%)	
**Diabetes Mellitus**			< 0.001
**Yes**	160 (36.61%)	277 (63.39%)	
**No**	733 (15.11%)	4119 (84.89%)	

^*^Resulted from independent samples t-test or chi-squared test

The evaluation metrics of different classification models under the default cut-point, and the optimum cut-point recommended by the precision-recall curve are presented in Table 4.

**Table 4:** Evaluation metrics percentage of different models under default and precision-recall curve cut-points

**Model**	**Cut-Point**	**Accuracy**	**nMCC**	**Balanced Accuracy**	**Sensitivity**	**Specificity**	**AUROC/AUPRC**
**BARTm 90%**	Default	69.48 ± 0.28	60.09 ± 0.81	63.12 ± 0.78	54.00 ± 1.66	72.23 ± 0.33	68.88 ± 0.40
	PRC	69.34 ± 4.83	60.41 ± 0.79	63.60 ± 0.83	55.37 ± 8.21	71.83 ± 7.13	27.71 ± 0.69
**BARTm 10%**	Default	70.03 ± 0.57	59.63 ± 0.92	62.38 ± 0.81	51.43 ± 1.44	73.33 ± 0.59	67.97 ± 0.77
	PRC	67.68 ± 3.75	59.98 ± 0.73	63.26 ± 0.71	56.93 ± 6.05	69.58 ± 5.46	27.18 ± 0.81
**LR**	Default	64.75 ± 0.23	62.35 ± 0.17	67.00 ± 0.18	70.23 ± 0.42	63.78 ± 0.30	73.37 ± 0.07
	PRC	74.98 ± 1.02	63.11 ± 0.18	66.08 ± 0.29	53.32 ± 2.09	78.83 ± 1.56	34.44 ± 0.14
**LDA**	Default	65.06 ± 0.23	62.19 ± 0.18	66.74 ± 0.19	69.14 ± 0.43	64.33 ± 0.30	73.28 ± 0.07
	PRC	74.50 ± 1.20	63.03 ± 0.14	66.11 ± 0.34	54.10 ± 2.49	78.13 ± 1.85	34.28 ± 0.12
**QDA**	Default	75.50 ± 0.08	62.45 ± 0.16	64.95 ± 0.13	49.84 ± 0.25	80.06 ± 0.09	72.10 ± 0.06
	PRC	74.55 ± 1.37	62.57 ± 0.07	65.45 ± 0.34	52.42 ± 2.79	78.47 ± 2.10	29.62 ± 0.11
**kNN**	Default	63.83 ± 0.33	59.87 ± 0.36	63.52 ± 0.38	63.07 ± 0.72	63.96 ± 0.39	67.94 ± 0.37
	PRC	64.65 ± 1.09	59.93 ± 0.33	63.53 ± 0.39	61.92 ± 2.05	65.14 ± 1.62	27.01 ± 0.41
**SVM**	Default	62.04 ± 0.24	62.02 ± 0.17	66.71 ± 0.20	73.39 ± 0.44	60.03 ± 0.31	66.62 ± 1.43
	PRC	62.75 ± 2.42	60.01 ± 1.11	63.80 ± 1.13	65.31 ± 3.67	62.30 ± 3.34	10.90 ± 0.32
**DT**	Default	51.95 ± 0.69	60.52 ± 0.23	64.52 ± 0.33	82.52 ± 1.22	46.52 ± 0.98	64.74 ± 0.58
	PRC	53.86 ± 2.38	60.39 ± 0.22	64.45 ± 0.36	79.60 ± 3.96	49.30 ± 3.50	20.76 ± 0.67
**RF**	Default	65.28 ± 0.27	60.82 ± 0.36	64.74 ± 0.39	63.97 ± 0.82	65.51 ± 0.33	69.44 ± 0.23
	PRC	62.13 ± 1.73	61.61 ± 0.24	66.12 ± 0.39	71.83 ± 3.16	60.41 ± 2.59	25.39 ± 0.36
**BART**	Default	61.83 ± 0.49	60.65 ± 0.45	64.77 ± 0.51	68.99 ± 1.07	60.56 ± 0.62	70.10 ± 0.41
	PRC	72.45 ± 4.35	61.30 ± 0.52	64.22 ± 0.85	52.44 ± 8.02	76.00 ± 6.54	30.69 ± 0.61
**ANN**	Default	62.85 ± 0.21	62.24 ± 0.16	66.98 ± 0.17	72.90 ± 0.37	61.06 ± 0.27	73.35 ± 0.08
	PRC	72.51 ± 1.25	63.13 ± 0.18	66.84 ± 0.33	58.70 ± 2.51	74.97 ± 1.91	33.91 ± 0.32
**GBM**	Default	61.99 ± 0.28	61.67 ± 0.25	66.22 ± 0.30	72.26 ± 0.68	60.17 ± 0.37	72.26 ± 0.16
	PRC	77.56 ± 2.62	62.80 ± 0.34	64.64 ± 0.59	46.14 ± 5.09	83.14 ± 3.98	33.65 ± 0.36

**Fig. 2:**
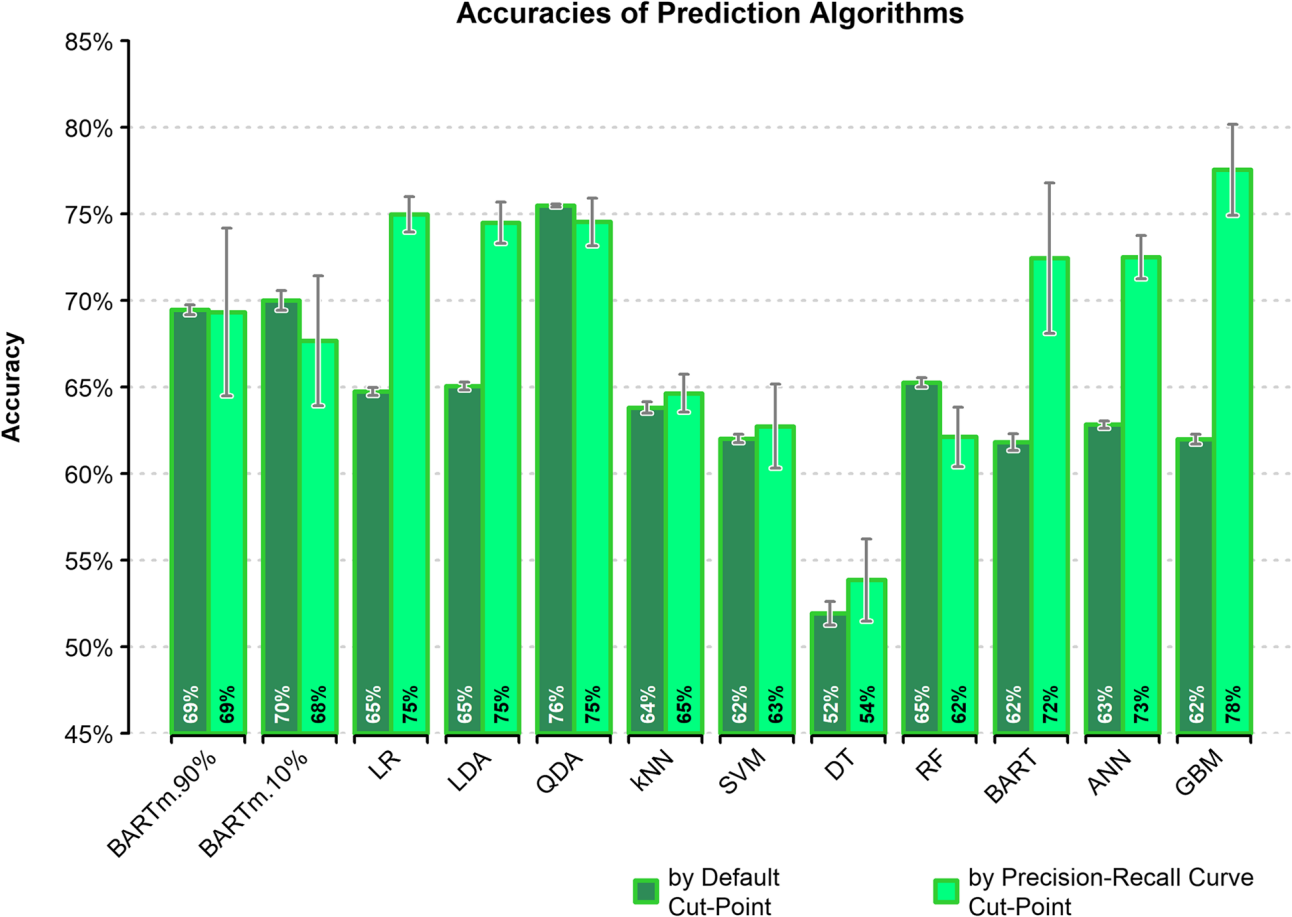
Accuracies of different models with corresponding SD

Figure 2 presents the mean accuracies (as percentage) along with SD as error bars of used prediction models.

**Fig. 3:**
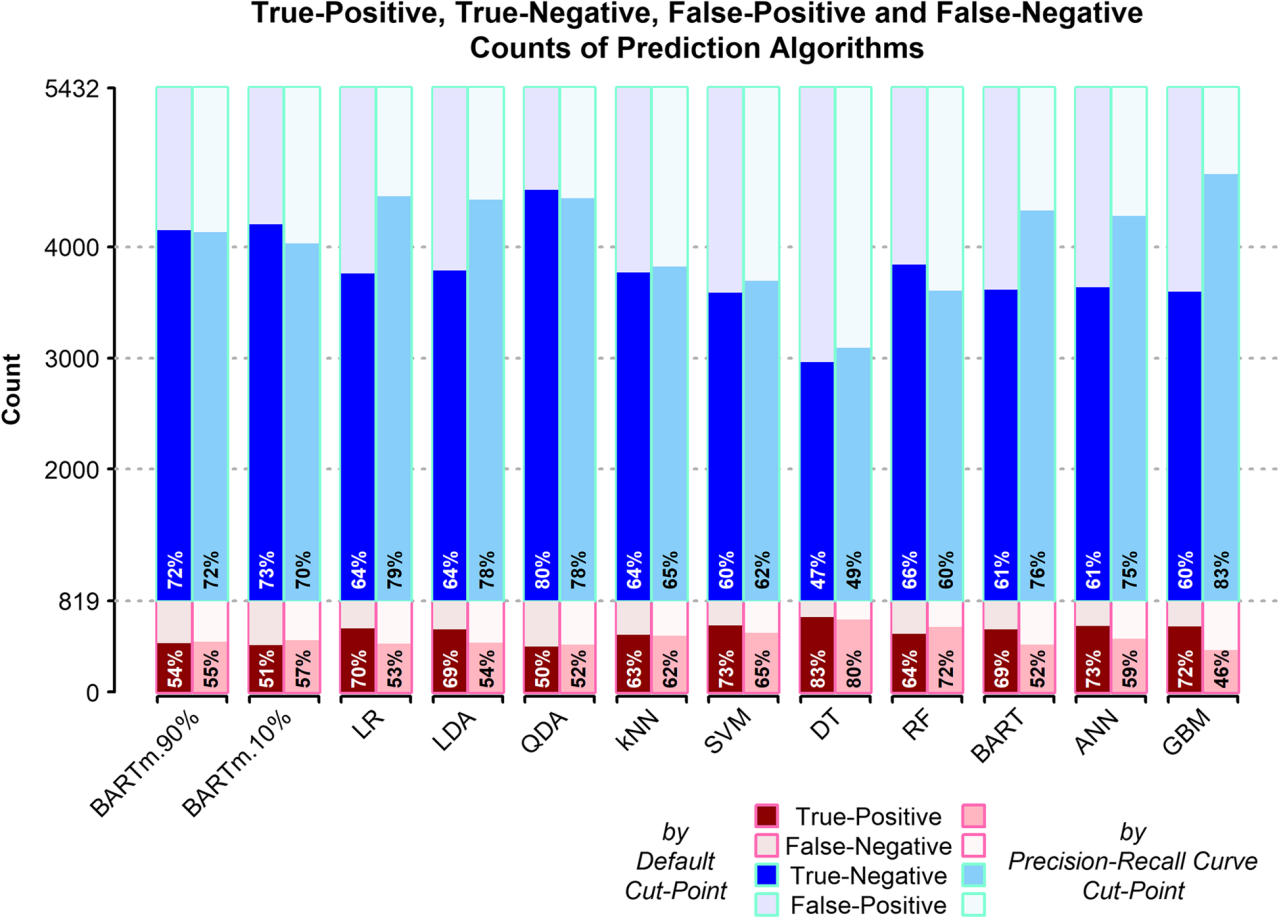
True-positive, false-negative, true-negative, and false-positive values of different prediction algorithms

Figure 3 shows true-positive, false-negative, true-negative, and false-positive values obtained from applying 12 different prediction models and two scenarios considered for cut-points. Sensitivity (proportion of correctly predicted CVD among participants who catch CVD) and specificity (proportion of non-CVD predicted participants among the participants who do not get CVD) are displayed in red and blue bars, respectively.

According to the default cut-point, QDA presents the highest prediction accuracy (75.50%), and DT provides the lowest accuracy (51.95%). Followed by QDA, BARTm.10% and BARTm.90% showed the highest accuracy with values of 70.03% and 69.48%, respectively. On the other hand, DT showed the highest sensitivity (82.52%); While QDA, BARTm.10%, and BARTm.90% showed the lowest sensitivity with values of 49.84, 51.43, and 54.00%, respectively, which is in the opposite flow of accuracy.

LR and ANN models by default cut-point produce the highest AUROC (73.37 and 73.35%, respectively) and the highest balanced accuracy (67.00 and 66.98%, respectively). The DT model based on default cut-point produces the lowest AUROC (64.74%), and BARTm.10% by default cut-point provides the lowest balanced accuracy (62.38%). According to MCC, ANN, LR, and LDA based on precision-recall curve cut-point resulted in the highest, almost the same, values of 63.13, 63.11, and 63.03%, respectively.

Generally, across the majority of accuracy, sensitivity, and specificity, QDA showed the best predictive performance. While across the majority of AUROC, balanced accuracy, and MCC, LR and ANN showed the best performance. Overall, DT had the weakest performance.

Using the precision-recall curve recommended cut-point instead of the default cut-point led to obtaining higher sensitivity and lower accuracy in all used models except for BARTm, QDA, and RF algorithms. For the GBM model, changing the default cut-point led to a 26.12% increase in sensitivity and a 15.57% decrease in accuracy. Another difference between the default cut-point and precision-recall curve cut-point is related to the SDs of metrics; In all models, the default cut-point produces smaller SD for accuracy, sensitivity, and specificity. According to the bias-variance trade-off, the higher accuracy and lower SD derived from changing the cut-point leads to the conclusion that using the recommended cut-point of the precision-recall curve causes more overfitting.

**Fig. 4:**
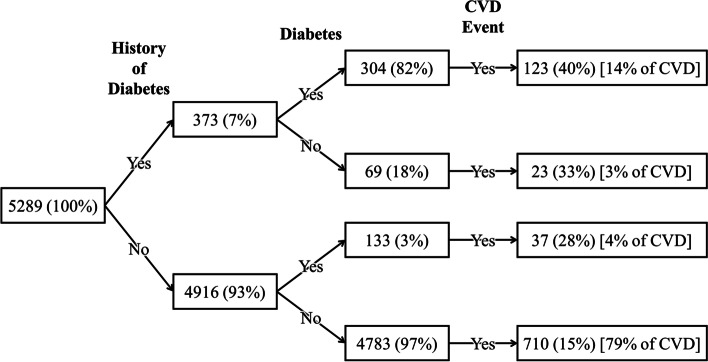
Flow of history of diabetes, diabetes, and occurrence of CVD events status

The RFE procedure revealed that diabetes and the history of diabetes have different effects on the occurrence of CVD events. Figure 4 shows the flow of history of diabetes, diabetes, and CVD events. Positive history of diabetes covers 17 (14 + 3) percent of CVD events, and adding diabetes status to the history of diabetes causes 4% more coverage of CVD events.

## Discussion

In this study, we used popular ML algorithms alongside conventional statistics models to predict the occurrence of CVD events at an early stage from a cohort study from the eastern Mediterranean region. The results revealed that only eight baseline variables were able to predict future CVD events accurately. So, by taking advantage of these techniques, primary prevention of CVD can be simple and cost-effective. Generally, the QDA algorithm produces accurate and stable predictions even with the default classification cut-point. By adopting the precision-recall curve’s recommended cut-point, GBM, LR, LDA, and ANN algorithms produce considerably higher prediction power.

ML techniques have become a practical tool in CVD prediction [19]. Dinh et al. used the National Health and Nutrition Examination Survey (NHANES) dataset to predict CVD; age, systolic blood pressure, self-reported weight, chest pain occurrence, and diastolic blood pressure were selected as the most contributing predictors. They achieved 83.9% AUROC with their developed ensemble model [48]. Mandair et al. used harmonized Electronic Health Record (EHR) data to predict myocardial infarction; While the AUC of deep neural network (83.5%) was quite similar to logistic regression (82.9%), they suggest that deep neural network algorithm may not offer substantial benefit compared to traditional logistic regression model using established risk factors [49].

Despite the presence of well-known prediction algorithms such as the Framingham heart study and European Systematic Coronary Risk Evaluation (SCORE) [50, 51], it is beneficial to investigate the risk factors in each region separately. Due to the main differences in intrinsic discrepancy, social environment, lifestyle, and genetic predisposition can cause different contributing factors and behavior. In this study, the most contributing variables for CVD prediction were identified as age, SBP, FBS, two-hour postprandial glucose, diabetes mellitus, history of heart disease, history of high blood pressure, and history of diabetes. These variables were confirmed by validated CVD risk prediction tools such as the joint guideline of American College of Cardiology (ACC) and American Heart Association (AHA) [52], the Framingham heart study, and SCORE [50, 51]. The common risk factors in the ACC/AHA guideline are gender, age, total cholesterol, HDL, smoking status, SBP, and diabetes [52]. Framingham heart study and SCORE refer to age, serum levels of lipids, especially HDL, smoking, diabetes mellitus, and blood pressure as risk factors for cerebrovascular disease, peripheral artery disease, heart failure, and coronary heart disease [50].

Age is considered the most contributing risk factor for CVD [52]. Hypertension, especially high SBP, was pathologically related to CVD and its risk factors like dyslipidemia and insulin resistance [53]. Studies have already shown that primary CVD event is a risk factor for the recurrence of CVD [54]. In this study, previous heart disease, as a part of medical examination check-up data, was selected as a contributing variable for CVD prediction. In this study, smoking was not selected as a prognosis variable. However, smoking status is known as a CVD risk factor [3, 5–7], especially in peripheral artery disease [55]. In this region, the prevalence of females who smoke is very low (2.2%), and approximately half of this study population contains females (51.0%); While in Europe and other developed countries, females smoke nearly as much as men [56]. Therefore, maybe the low prevalence of females who smoke in this region is the reason for not seeing smoking status as a contributing variable for CVD prediction. HDL is famous as “good cholesterol”, so low levels of HDL are known as a CVD risk factor [15, 57]. However, some studies failed to prove the prevention effect of controlling the levels of HDL on CVD events [58, 59]. So HDL is not necessarily causally associated with CVD, but normal HDL will not guarantee free CVD events [60]. In this context, HDL could be an indirect or surrogate variable that does not participate directly in causing CVD events [8, 61]. It should also be noted that the feature selection was done without including any knowledge about the clinical aspect of any variable; This can also be the reason for not selecting other CVD’s well-known risk factors by the RFE procedure.

The flow of Fig. 4 indicates that adding diabetes status to the history of diabetes causes 4% more coverage of CVD events. This 4% percent may be negligible, but the low prevalence of total CVD events makes it valuable. Since these two variables contain complementing information about events, the RFE procedure selects them correctly.

The BARTm’s combined algorithm makes it an effective and efficient algorithm. BARTm can accurately predict CVD incidence without any preprocessing, imputation, and feature selection steps. Also, it is applicable to all researchers without any need for technical knowledge of assumptions and preprocessing procedures of prediction models on large and even incomplete datasets [33, 62, 63].

None of the evaluation metrics, on their own, are enough to characterize the model performance. In this study, because of imbalanced CVD events, the model with higher accuracy has a higher specificity and vice versa. If a model predicts a non-CVD situation for all samples, specificity will be 100%, and accuracy will be 84.92%, but sensitivity will be 0%; Although it is also necessary to correctly predict CVD events. So, in this study, having an acceptable sensitivity (at least 50%) and higher prediction accuracy was the criterion for selecting the best prediction model. Another approach is to consider balanced accuracy and MCC metrics that are more suitable for rare event situations and will consider both sensitivity and specificity [64].

ML techniques can reduce the variables of large datasets so that conventional statistical models can be applied. Unlike the complicated ML procedure, which is famous for black-box, simple models like LR and DT have their benefits. LR presents an odds ratio measure for any predictor, which is very helpful for interpretation. The DT model also provides a simple diagram to classify the samples by their specifications.

## Strengths and limitations

This study performed various prediction models using different packages on a large primary care cohort study with a 16-years follow-up period from a developing country. Compared to developed countries, the number of CVD research with a high-quality dataset in developing countries is still low due to funding limitations [27]. So even with the expected result as a clinical aspect, it can be novel and applicable in this region. Furthermore, the adoption of prediction approaches in each geographical region is more individualized, which can result in better risk assessment. In this study, 385 variables were entered, and only 8 of them were selected as the most contributing variables for prediction without involving any prejudice about risk factors. So, their well-known relation to the response variable will validate the feature selection procedure. Using a single laboratory and team for gathering the data in all follow-up periods is another strength of this study.

Loss to follow-up is a limitation of the study that belongs to the nature of cohort studies. Another limitation is the absence of HbA1c, three enzyme alleles ABO^A^, ABO^B^, and ABO^O^; Certainly, including such variables could improve the prediction accuracy. Additionally, the high level of missing values in the dataset can cause biases; Although the BARTm algorithm can address this issue, the complete dataset can lead to a more accurate result. More hyper-parameters in ML algorithms could be considered, but the models already achieved appropriate performance, so hyper-parameters had been covered in the grid search process. Generally, these limitations are unlikely to change our conclusion about the advantages of both ML and statistical models in CVD prediction. Because of the study inclusion criteria, there are two cautions which should be considered: (I) results derived from individuals with no CVD; (II) young individuals (age < 35), careful attention is needed.

## Conclusion

While CVD can be prevented by controlling some behavioral habits like a sedentary lifestyle, unhealthy diet, and smoking, the effective prediction models in each region can be beneficial to guide policymakers for screening programs and primary prevention of CVD. In this study, age, SBP, FBS, two-hour postprandial glucose, diabetes mellitus, history of heart disease, history of high blood pressure, and history of diabetes were the most contributing factors for predicting CVD events. Also, it is possible to accurately predict the occurrence of CVD events only with eight variables 16 years earlier.

Using the precision-recall curve recommended cut-point instead of the default cut-point increased sensitivity and decreased accuracy for all classification algorithms except for BARTm, QDA, and RF. Generally, based on accuracy, sensitivity, and specificity, QDA showed the best predictive performance. While based on AUROC, balanced accuracy, and MCC, LR and ANN showed the best performance. Overall, DT had the weakest performance. Researchers can use BARTm without the need for any technical knowledge of assumptions and preprocessing steps of prediction models on large and even incomplete datasets.

## Data Availability

The ICS datasets cannot be shared publicly due to ethical reasons, but are available from the Isfahan cardiovascular research institute for researchers on reasonable request. A representative deidentified of it is available from the figshare database (https://doi.org/10.6084/m9.figshare.5480224).

## Abbreviations

ACC: American college of cardiology
AHA: American heart association
AUPRC: Area under the precision-recall curve
AUROC: Area under the receiver operating characteristic curve
ANN: Artificial neural network
BART: Bayesian adaptive regression trees
BMI: Body mass index
CVD: Cardiovascular disease
DT: Decision trees
DBP: Diastolic blood pressure
FBS: Fasting blood sugar
GBM: Gradient boosting machine
HDL: High-density lipoprotein
ICRC: Isfahan cardiovascular research center
ICS: Isfahan cohort study
kNN: K-nearest neighbors
LDA: Linear discriminant analysis
LR: Logistic regression
LDL: Low-density lipoprotein
ML: Machine learning
BARTm: Missing incorporated in attributes within Bayesian additive regression trees
nMCC: Normalized Matthews correlation coefficient
PRC: Precision-recall curve
QDA: Quadratic discriminant analysis
RF: Random forest
RFE: Recursive feature elimination
SD: Standard deviation
SVM: Support vector machine
SCORE: Systematic coronary risk evaluation
SBP: Systolic blood pressure
WHO: World health organization
